# Effects of chronic lithium administration on renal acid excretion in humans and rats

**DOI:** 10.14814/phy2.12242

**Published:** 2014-12-11

**Authors:** I. David Weiner, John P. Leader, Jennifer J. Bedford, Jill W. Verlander, Gaye Ellis, Priyakshi Kalita, Frederiek Vos, Sylvia de Jong, Robert J. Walker

**Affiliations:** 1 Nephrology and Hypertension Section, NF/SGVHS, Gainesville, Florida; 2 Department of Medicine, University of Florida College of Medicine, Gainesville, Florida; 3 Department of Medicine, University of Otago, Dunedin, New Zealand

**Keywords:** Acid–base, ammonia, citrate, collecting duct, lithium

## Abstract

Lithium therapy's most common side effects affecting the kidney are nephrogenic diabetes insipidus (NDI) and chronic kidney disease. Lithium may also induce a distal renal tubular acidosis. This study investigated the effect of chronic lithium exposure on renal acid–base homeostasis, with emphasis on ammonia and citrate excretion. We compared 11 individuals on long‐term lithium therapy with six healthy individuals. Under basal conditions, lithium‐treated individuals excreted significantly more urinary ammonia than did control subjects. Following an acute acid load, urinary ammonia excretion increased approximately twofold above basal rates in both lithium‐treated and control humans. There were no significant differences between lithium‐treated and control subjects in urinary pH or urinary citrate excretion. To elucidate possible mechanisms, rats were randomized to diets containing lithium or regular diet for 6 months. Similar to humans, basal ammonia excretion was significantly higher in lithium‐treated rats; in addition, urinary citrate excretion was also significantly greater. There were no differences in urinary pH. Expression of the critical ammonia transporter, Rhesus C Glycoprotein (Rhcg), was substantially greater in lithium‐treated rats than in control rats. We conclude that chronic lithium exposure increases renal ammonia excretion through mechanisms independent of urinary pH and likely to involve increased collecting duct ammonia secretion via the ammonia transporter, Rhcg.

## Abstract (precis)

This study investigated the effect of chronic lithium exposure on renal acid–base homeostasis, with emphasis on ammonia and citrate excretion. Chronic lithium exposure increases renal ammonia excretion through mechanisms independent of urinary pH and likely to involve increased collecting duct ammonia secretion via the ammonia transporter, Rhcg.

## Introduction

Following their serendipitous demonstration as an effective treatment for bipolar disorder (Cade 1949), lithium salts have been widely prescribed for mood disorders. It has been estimated that about one in 1000 of the U.S. population has been prescribed, at some time or another, lithium salts for controlling mental disorders (Okusa and Crystal 1994; Marples et al. 1995). Acute and chronic lithium exposure in both rats and humans has long been known to cause changes, both structural and functional, in the kidney, many of which have been well documented (Grunfeld and Rossier 2009).

Lithium salts have significant effects on renal function ranging from mild impairment of urinary concentrating ability to full‐blown nephrogenic diabetes insipidus (NDI) (Boton et al. 1987). Mechanisms underlying the development of NDI have been well studied (Bichet 2006; Robben et al. 2006; Trepiccione and Christensen 2010). Less well documented are lithium‐induced changes in acid–base regulation. Earlier studies have shown that lithium can increase baseline urine pH (Perez et al. 1975; Miller et al. 1979), can impair generation of the urine‐blood Pco_2_ differences (Roscoe et al. 1976; Perez et al. 1977), and can impair acidosis‐induced changes in urine acidification in humans (Perez et al. 1975, 1977; Miller et al. 1979). However, assessment of urine pH and urine‐blood Pco_2_ differences are, at best, indirect measures of renal net acid excretion, and none of the studies listed above quantified urinary ammonia excretion, the predominant component of renal net acid excretion (DuBose et al. 1991).

The influence of chronic metabolic acidosis in the progression of chronic kidney disease has received increased attention recently. Because chronic lithium exposure, even at therapeutic levels, can lead to the development of interstitial fibrosis and chronic kidney disease, there is the potential that lithium‐induced abnormal acid–base regulation could contribute to the progression of lithium‐induced renal impairment. Accordingly, the purpose of the current studies was to determine the effect of chronic lithium exposure on acid–base regulation. We studied human subjects on long‐term chronic lithium therapy for their underlying psychiatric disorders. Urine pH and urinary ammonia excretion, both under baseline conditions and following an acute acid load, was compared to that observed in normal control subjects. We also studied rats treated for 6 months with lithium chloride addition to their diet. In rats, we examined the effect of chronic lithium administration on changes in the expression of the key ammonia transporter family member, Rhesus C Glycoprotein (Rhcg). Our results show that chronic lithium administration increases urine ammonia excretion and the ability to increase ammonia excretion in response to an acute acid load is maintained. Moreover, these effects are associated with significant increases in the expression of the key ammonia transporting protein, Rhcg. In rats, but not humans, chronic lithium exposure is also associated with increased citrate excretion.

## Materials and Methods

### Human studies

#### Participants

In this study, 11 participants (eight females, three males) on chronic lithium therapy for the management of their mood disorder were matched with six healthy volunteers (three females, three males). Participants were recruited from a cohort of individuals on long‐term lithium therapy who have previously participated in our clinical studies (Bedford et al. 2008). Inclusion criteria included individuals with bipolar disorder treated with lithium carbonate, who were clinically stable, with no change in their medications over the preceding 3 months, and who had no known history of renal disease. Exclusion criteria included the inability to give informed consent, a history of known renal disease, the continued use of a diuretic or angiotensin‐converting enzyme inhibitor, unstable psychiatric condition, or recent changes in psychotropic medications. All participants gave written informed consent to take part in the study, which was approved by the New Zealand (Lower South Regional) Ethics Committee.

#### Clinical studies

Subjects presented to the Clinical Research Area (Department of Medicine, Dunedin School of Medicine). Height and weight were recorded. Participants were instructed to take their routine medications following baseline blood samples on the day of study. Baseline blood samples for plasma pH, bicarbonate, chloride, sodium, potassium, and creatinine were taken. Baseline urine samples were collected, and analyzed for pH, potassium, sodium, chloride, lithium, creatinine, citrate (BioVision, Milptes, CA), and total ammonia (Pointe Scientific, Canton, MI). Participants then received a dose of ammonium chloride (100 mg/kg [1.87 mmol/kg] body weight) in capsular form (each capsule contained 500 mg ammonium chloride), but this was limited to a maximum of 14 capsules/person. Blood samples were collected prior to and at 2, 4, and 6 h following the dose, and all urine was collected prior to and at 2, 4, and 6 h after the ammonium chloride dose. Plasma pH, chloride, bicarbonate, sodium potassium, and creatinine as well as urinary pH, sodium, potassium, chloride, and creatinine were measured by standard automated laboratory assays (Healthlab Otago, Dunedin Hospital, New Zealand).

### Animal studies

The animal studies were approved by the University of Otago Animal Ethics Committee (92/08) under New Zealand National Animal Welfare guidelines. We used 32 Wistar male rats (200 g) from the Hercus‐Taieri Resource Unit, University of Otago, which were separated randomly into two groups. Sixteen were subjected to a diet containing lithium, so as to induce nephrogenic diabetes insipidus (NDI), while the others were used as controls.

To induce NDI, the protocol of Kwon et al. (2000) was followed. Rats were given access to standard rodent diet (Speciality Foods, Perth, Australia, meat‐free diet) containing 40 mmol/L lithium/kg dry‐powdered food for the first 7 days, followed by 60 mmol/L lithium/kg dry food for the remaining 25 weeks. Tap water was supplied ad libitum. To ensure adequate mixing, the food pellets were ground to a very fine powder. It was found (Kairuz et al. 2005) that this protocol resulted in plasma lithium levels comparable to the therapeutic levels in human plasma (0.8 – 1.3 mmol/L) and minimized the slower than normal gain of weight often caused by lithium treatment. All rats administered lithium were given access to a salt block to maintain sodium balance and prevent lithium intoxication. Control rats received the same diet, but without the lithium. Body weight was measured weekly and water intake was measured every second day.

After 6 months on the lithium regime, the experimental rats, together with an equal number of controls, were transferred individually to metabolic cages, where water intake could be monitored, and urine collected under water‐saturated mineral oil over 24 h. A blood sample (100 μL) was removed from the tail (mixed venous/arterial) of unanesthetized rats and pH, Po_2_, Pco_2_ measured in a blood gas analyzer (Radiometer NPT series 7, Copenhagen, Denmark). Urine volume and composition (pH, sodium, potassium, lithium, chloride, and creatinine concentrations) were determined. Water uptake was monitored over the same time periods.

At the conclusion of the experimental period, all rats were euthanized by decapitation after carbon dioxide narcosis, and a blood sample was collected, spun immediately, and the plasma removed. Both kidneys were rapidly excised and cut in half longitudinally. Half of one kidney was fixed in 10% buffered formol saline for microscopy. The remaining three halves were snap frozen in liquid nitrogen and stored at −80°C for later analysis.

### Biochemical analyses

The osmolality of urine and plasma samples from rats was determined using a vapor pressure osmometer (Wescor 5500, Logan, UT). Urinary pH was measured and sodium, lithium, and potassium concentrations in the urine and plasma were determined by flame photometry (Radiometer FLM3, Copenhagen, Denmark). Chloride in the plasma was determined electrometrically using a Cotlove chloride titrator. Plasma BUN (Pointe Scientific) was measured in the plasma, and creatinine concentrations in urine and plasma were measured, after appropriate dilution (Randox Laboratory Ltd, Antrim, UK), to confirm normal renal function. Citrate in the urine was measured colorimetrically (BioVision) and ammonia determined using a Pointe Scientific kit (A7553) modified for small volumes (Lee et al. 2009). Urinary titratable acid was measured using the method of Chan (Chan 1972) modified to use 25 or 50 μL of urine (Lee et al. 2009). Briefly, samples were acidified by the addition of an equal volume of 0.1 mol/L HCl, boiled for 2 min, and then cooled to 37°C. The amount of 0.4 mol/L NaOH required to titrate the sample back to pH 7.40 was measured. Samples of deionized water were analyzed in parallel, and results were subtracted from urine samples to yield net titratable acid. Net acid excretion was calculated as the sum of urinary ammonia and titratable acid excretion.

### Microscopy

For histological examination, kidneys were wax embedded and sections cut at 3 μm. After rehydration, sections were stained with Masson's Trichrome, cleared in xylol, and mounted in DPX. For immunohistochemistry antigen retrieval was carried out (microwave 10 min in 10 mmol/L citrate buffer at pH 6.0), followed by blocking of endogenous peroxidase activity with 3% H_2_O_2_ in PBS. The sections were then preincubated in 1% BSA (Sigma‐Aldrich, St Louis, MO) in PBS to block nonspecific binding. They were then labeled with the appropriate primary antibody. Antibodies used were against Rhcg and Rhbg (Verlander et al. 2003), and were visualized using a horseradish peroxidase‐coupled secondary antibody (goat anti‐rabbit IgG, (DAKO, Glostrup, Denmark), followed by incubation with diaminobenzidine (Sigma‐Aldrich). After dehydration and clearing, sections were mounted in DPX. They were viewed using a Provis AX 70 Olympus microscope, and images of representative regions were recorded using a SPOT digital camera attached to a Macintosh computer running SPOT proprietary software. Later examination and analysis of the images were performed using ImageJ (National Institutes of Health, Bethesda, MD). Negative controls were carried out either by omitting the primary antibodies and/or by using appropriate blocking peptides.

### Western blotting

Samples from the cortex, and inner and outer medulla were homogenized in buffer (0.3 mol/L sucrose, 0.25 mmol/L imidazole, 1 mmol/L EDTA containing protease inhibitors leupeptin (8.5 μmol/L), phenylmethylsulphonyl fluoride (PMSF) (1 mmol/L), and 1% SDS). They were centrifuged (5000 g for 15 min at 4°C) to remove cellular debris and total protein measured using BCA reagent (Pierce Chemical Co., Thermo Fisher Scientific, Rockford, IL). Laemmli buffer was added in the ratio of 1: 2. The samples were separated on 12% SDS premade TGX polyacrylamide gels (BioRad, Hercules, CA) and electroblotted onto Immobilon PDVF membranes (Millipore, Billerica, MA). Loading and transfer equivalence were assessed by staining with Ponceau S. The membranes were blocked with 5% nonfat milk in buffer (25 mmol/L tris, pH 7.6) containing 1% Tween 20 for 1 h and then incubated with the appropriate primary antibody. They were then washed, reacted with the secondary antibody (goat anti‐rabbit IgG‐HRP, DAKO), and visualized by chemiluminescence using Supersignal Pico West (Pierce Chemical Co., Thermo Fisher Scientific). X‐ray films were scanned using a BioRad (GS‐700) densitometer using Quantity One, version 4.3.2 (BioRad) software.

### Statistics

Quantitative results are expressed as means ± SEM. Differences among the means of multiple parameters were analyzed by ANOVA followed by the Student–Newman–Keuls test in Kaleidograph (Synergy software; Synergy Si Inc., Canberly, UK). Values of *P *<**0.05 were considered statistically significant.

## Results

### Human studies

Participants' demographic data are presented in Table 1. Plasma lithium concentrations were within the accepted therapeutic range in all participants (0.46–1.04 mmol/L) (Table 1).

**Table 1.** Demographic data of all human participants.

Group	Gender (M:F)	Age	Time on lithium (years)	Plasma lithium (mmol/L)
Lithium‐treated	3:8	63±3 (47–74)	20 ± 3 (5–38)	0.63 ± 0.06 (0.46–1.04)
Control	3:3	44±4 (28–55)	N/A	N/A

N/A, not applicable.

Baseline plasma and urinary measurements findings are shown in Table 2. Plasma electrolytes, including sodium, potassium, chloride, and bicarbonate, did not differ significantly between lithium‐treated and control participants. As expected, urine osmolality was significantly lower in lithium‐treated participants than in control participants, consistent with the well‐known effect of lithium to cause development of a partial NDI. The human subjects in the lithium arm fell into three groups with respect to their ability to concentrate urine. Of the 11 subjects, 10 showed partial NDI, with a urine osmolality of between 750 and 300 mosm/kg water. Three of them had urine osmolalities between 300 and 400 mosm/kg water. The remaining subject had a urine osmolality of 815 mosm/kg water.

**Table 2.** Baseline plasma and urinary parameters in humans.

	Control	Lithium	*P* Value
Plasma			
Na^+^, mmol/L	140.3 ± 0.8	139.8 ± 0.8	NS
K^+^, mmol/L	4.4 ± 0.1	4.1 ± 0.1	NS
Cl^−^, mmol/L	102.7 ± 0.4	105.4 ± 0.7	NS
HCO_3_^−^, mmol/L	26.8 ± 0.1	28.0 ± 0.7	NS
Urine			
Osmolality, mOsm/kg H_2_O	710 ± 86	478 ± 61	<0.05
pH	7.02 ± 0.35	6.91 ± 0.24	NS
Ammonia, mmol/mmol creatinine, [mmol/g creatinine]	3.18 ± 0.97 [28.1 ± 8.5]	9.41 ± 2.23 [83.2 ± 20.7]	<0.05
Titratable Acid, mmol/L/mmol/L creatinine, [mmol/g creatinine]	1.33 ± 2.29 [11.7 ± 20.2]	4.46 ± 1.20 [39.4 ± 10.6]	NS
Citrate, μmol/L/mmol/L creatinine, [μmol/g creatinine]	58.5 ± 22.8 [517.0 ± 201.9]	60.3 ± 12.2 [533 ± 108.2]	NS
Net acid excretion, mmol/L/mmol/L creatinine, [mmol/g creatinine]	4.78 ± 3.32 [42.2 ± 28.6]	14.39 ± 3.65 [127.2 ± 32.2]	<0.05

Significant differences were found between the groups with respect to urinary acid excretion (Table 2). In particular, urinary ammonia excretion was significantly higher in lithium‐treated participants than in control participants, averaging threefold greater. Although urine pH was slightly lower in lithium‐treated participants, this did not reach statistical significance. Although a lower urinary pH can increase renal ammonia excretion, there was no correlation of urine pH with urinary ammonia excretion (*P* = NS by ANOVA). Finally, urinary citrate excretion did not differ between lithium‐treated and control participants.

### Effect of lithium therapy in response to acid loading

**Figure 1.**
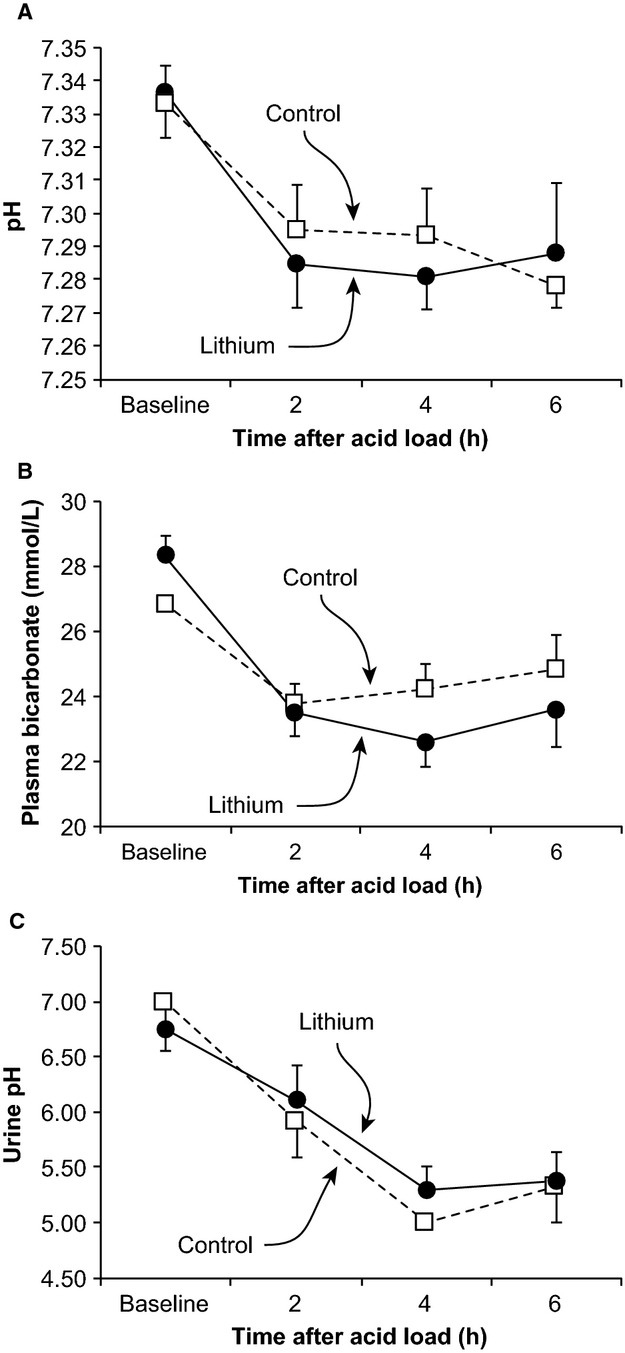
Effect of lithium therapy on systemic changes in urinary pH in response to an acute acid load in humans**.** Panel A shows arterialized pH responses to an acute acid load. There was no significant difference in arterialized pH between lithium‐treated and control subjects under baseline conditions. An acute acid load, induced by oral ammonium chloride loading, resulted in rapid and significant decreases in pH in both lithium‐treated and control subjects. At no time point, did the change in pH differ between lithium‐treated and control subjects. Panel B shows baseline and acute acid‐loading‐induced changes in serum bicarbonate concentration. There was no significant difference in either baseline serum bicarbonate or serum bicarbonate at any time point following acute acid loading between lithium‐treated and control subjects. Panel C shows urinary pH. Urinary pH was similar in lithium‐treated and control subjects at baseline. An acute acid load resulted in significant urinary acidification in both lithium‐treated and control subjects at each time point. There was no significant difference in either absolute urinary pH or decrease in response to an acid load between lithium‐treated and control subjects at any time point.

We next examined whether the chronic lithium treatment altered the ability to respond to an acute acid load. We used a standard oral ammonia chloride loading protocol. Figure 1 summarizes these results. Ingestion of an ammonium chloride acid load resulted in development of acute metabolic acidosis, whether measured as systemic pH or as plasma bicarbonate concentration, in both lithium‐treated and control subjects. At no time point, either baseline or following ingestion of the acid load, did either systemic pH or plasma bicarbonate differ significantly between lithium‐treated and control subjects. In addition, the magnitude of decrease from baseline of the plasma bicarbonate concentration did not differ at any time point between the two groups. Thus, neither baseline pH nor the development of acute metabolic acidosis in response to an oral ammonium chloride load differs between lithium and control subjects.

We then assessed the urinary pH response to the acid load. Under baseline conditions, urinary pH did not differ significantly between lithium‐treated and control subjects. Following ingestion of an acute acid load, urinary pH decreased, consistent with the normal renal response to increased plasma acidification. Urine pH did not differ significantly at any time point between lithium‐treated and control subjects. Thus, chronic lithium exposure did not impact the response to an acute acid load in terms of systemic acid–base changes or either baseline urinary pH or changes in urinary pH in response to the acid load.

**Figure 2.**
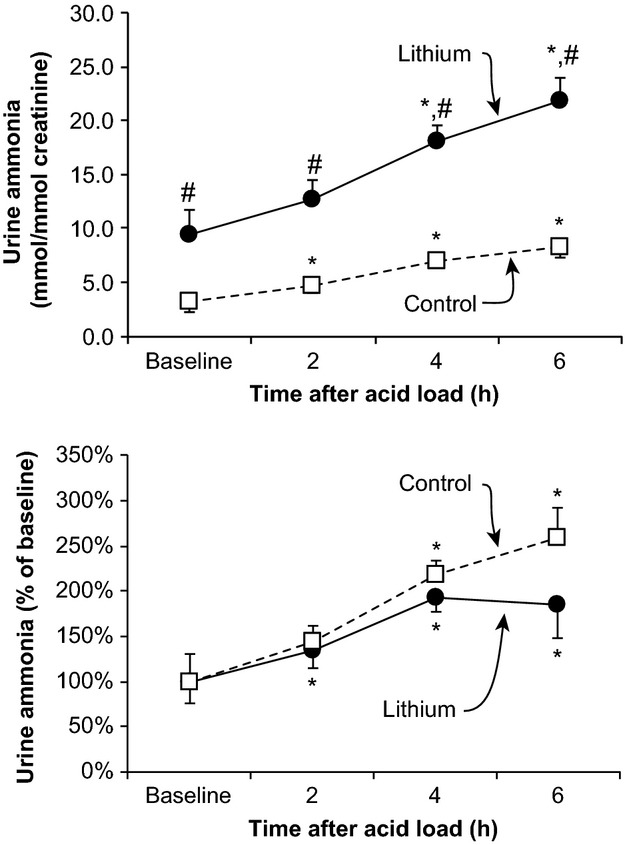
Effect of lithium therapy on urinary ammonia excretion in response to an acute acid load in humans. Left panel shows urinary ammonia excretion, expressed as millimoles of ammonia per millimole creatinine, at baseline, and following an acute acid load. Baseline urinary ammonia excretion was significantly higher in lithium‐treated subjects than in control subjects. An acute acid load, induced by oral ammonium chloride loading, resulted in significant increases in urinary ammonia excretion in both groups at each time point. However, at each time point, urinary ammonia excretion was significantly greater in lithium‐treated subjects than in control subjects. Bottom panel shows changes in urinary ammonia excretion adjusted for baseline urinary ammonia excretion rates. An acute acid load resulted in significant increases in ammonia excretion relative to baseline excretion in both groups. *P < 0.05 versus baseline; ^#^*P* < 0.05 versus control at same time point.

The quantitatively predominant mechanism by which the kidneys increase net acid excretion following an acute acid load is to increase urinary ammonia excretion (Elkinton et al. 1960; Star et al. 1987a). Figure 2 summarizes the effect of chronic lithium treatment on the renal excretion of ammonia in response to an acute acid load. As noted previously, baseline urinary ammonia excretion was significantly higher in lithium‐treated than in control participants. In both groups, acid loading increased renal ammonia excretion significantly. Because baseline ammonia excretion differed significantly, we also examined quantitatively the changes in ammonia excretion relative to baseline excretion rates. As shown in Figure 2, acute acid loading increased urinary ammonia excretion in both groups, but the magnitude of the increase in urinary ammonia excretion, relative to the basal rate of ammonia excretion, did not differ between lithium‐treated and control subjects. Thus, chronic lithium treatment increases baseline ammonia excretion and the response to an acute acid load in terms of ammonia excretion is maintained.

### Effect of lithium on citrate excretion in humans

**Figure 3.**
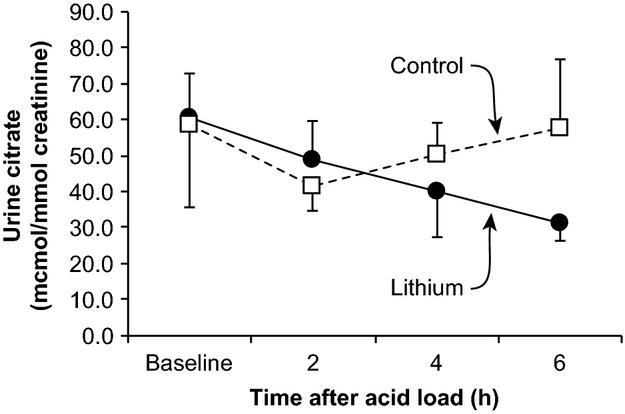
Effect of lithium treatment on urinary citrate excretion in humans. Baseline urinary citrate excretion was similar in lithium‐treated and control subjects. Following an acute acid load, there were no significant changes in urinary citrate excretion in either lithium‐treated or control subjects. Although there was a tendency for a progressive decrease in urinary citrate excretion following the acid load in lithium‐treated subjects, this change did not reach statistical significance. At no time following the acute acid load did urinary citrate excretion differ between lithium‐treated and control subjects.

Lithium treatment frequently increases plasma calcium and causes development of primary hyperparathyroidism (Franks et al. 1982; Grunfeld and Rossier 2009). Urinary citrate excretion can play an important role both in acid–base homeostasis and in prevention of calcium‐related nephrolithiasis (Moe and Preisig 2006; Weiner and Verlander 2012). Accordingly, we assessed the effect of lithium treatment on basal and acidosis‐stimulated urinary citrate excretion. Figure 3 summarizes these results. Urinary citrate excretion did not differ significantly between lithium‐treated and control subjects under baseline conditions. In response to acid loading, urinary citrate excretion did not change significantly over the 6‐h duration of these studies. In addition, at no time point, did urinary citrate excretion differ significantly between control and lithium‐treated subjects. These results indicate, overall, that acute acid loading has no effect on urinary citrate excretion, and that lithium treatment does not alter either baseline or acidosis‐stimulated citrate excretion.

### Rat studies

Rats fed the lithium diet rapidly developed, within 1 week, a copious diuresis that was maintained throughout the experimental period, consistent with the known effect of lithium to cause NDI. Urine output and composition after 6 months of the lithium diet are shown in Table 3. Urine osmolality in lithium‐treated animals was greatly reduced compared to controls (with a mean value, at 6 months of 176 ± 29 mosm/kg water compared with 1729 ± 428 mosm/kg water in urine from control animals) and the volume of urine produced increased about 10 fold, from 45 ± 6 (control) to 468 ± 41 μL/min/kg body weight. Analyses of plasma gave normal values for plasma osmotic pressure, sodium, potassium, and chloride concentrations. Plasma lithium averaged 0.92 ± 0.05 mmol/L in lithium‐treated rats, which is in the typical therapeutic range for lithium administration in humans.

**Table 3.** Physiological parameters of control and lithium‐fed rats.

	Control	Lithium	*P* Value
Plasma composition			
Na^+^, mmol/L	136 ± 1.7	140 ± 1.4	NS
K^+^, mmol/L	5.3 ± 0.2	5.4 ± 0.1	NS
Li^+^, mmol/L	ND	0.92 ± 0.05	
Cl^−^, mmol/L	94.3 ± 2.5	88.9 ± 1.6	0.037
pH	7.37 ± 0.02	7.39 ± 0.02	NS
HCO_3_^−^, mmol/L	26.7 ± 0.13	28.1 ± 1.6	NS
Osmolality, mosm/kg water	298 ± 3	295 ± 3	NS
Creatinine, μmol/L	93 ± 9	94 ± 4	NS
Urea (BUN), mmol/L	7.89 ± 0.65	7.39 ± 0.79	NS
Fluid balance			
Water intake, μL/min/kg	50 ± 7	552 ± 55	<0.001
Urine output, μL/min/kg	45 ± 6	468 ± 41	<0.001
Urine composition			
Na^+^, mmol/L	61 ± 9	11 ± 0.7	<0.001
K^+^, mmol/L	135 ± 14	12 ± 0.06	<0.001
Li^+^, mmol/L	ND	3.3 ± 0.26	<0.001
Osmotic pressure, mosm/kg water	1729 ± 428	176 ± 29	<0.001
Protein, mg/h/kg bw	16.2 ± 4.2	29.3 ± 4.1	NS
Urine acid‐base excretion			
pH (baseline)	7.78 ± 0.14	7.20 ± 0.14	NS
Ammonia, nmol/L/min/kg bw	481 ± 109	3963 ± 560	<0.001
Titratable acid, nmol/L/min/kg bw	−5.28 ± 1.59	66.39 ± 3.01	0.026
Citrate, nmol/L/min/kg bw	2.9 ± 0.8	142.4 ± 12.9	<0.001
Net acid excretion, nmol/L/min/kg bw	−2.60 ± 1.51	74.76 ± 30.50	<0.001

ND, not determined.

Values are mean ± SEM (*n* = 8 in each group).

Renal function, as assessed by plasma creatinine and urea levels, remained normal. The amount of protein in the urine increased slightly, but the results were not statistically significant, and did not reach the levels that would be expected from severe glomerular disease.

Acid–base parameters were similar in control and lithium‐treated rats. Plasma pH and bicarbonate did not differ significantly between control and lithium‐treated rats. However, urinary ammonia, titratable acid and citrate excretion increased markedly with lithium treatment. Thus, similar to humans, chronic lithium administration to rats results in significant increases in urinary ammonia excretion in the absence of a change in systemic acid–base balance or in urinary pH.

### Histology

**Figure 4.**
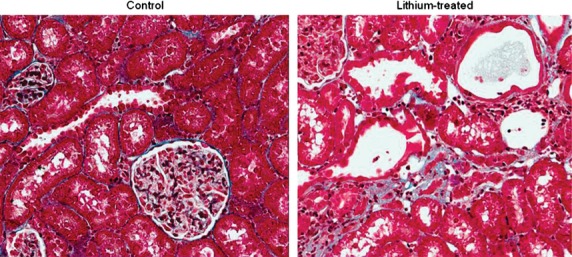
Masson's Trichrome staining of the kidneys of control (left panel) and lithium‐treated (right panel) rats. The histologic picture of control‐treated rats showed normal glomeruli, tubules, interstitium, and vasculature. There was no evidence of age‐related renal injury. In contrast, in the lithium‐treated animals substantial abnormalities were evident. There were large areas of local fibrotic lesion and accumulation of leukocytes. The epithelial lining of cortical collecting duct segments was variable in morphology, ranging from areas where the cells are greatly thinned to regions occupied by large, hypertrophic cells with enlarged nuclei.

Histological examination of thin sections of the kidneys of lithium‐fed animals revealed substantial abnormalities. In the renal cortex, distal regions of the tubules were focally dilated and there were large areas of focal fibrotic lesions and interstitial accumulations of leukocytes. The epithelial lining of the cortical collecting ducts was extremely variable in morphology, ranging from areas where the cells were greatly flattened against the basement membrane to regions occupied by large, distended cells with greatly enlarged nuclei (Fig. 4). The medullary collecting ducts appeared to have a greatly increased cell density compared to controls, but quantification was made difficult by the varied extent of tubular dilation.

### Western blotting

**Figure 5.**
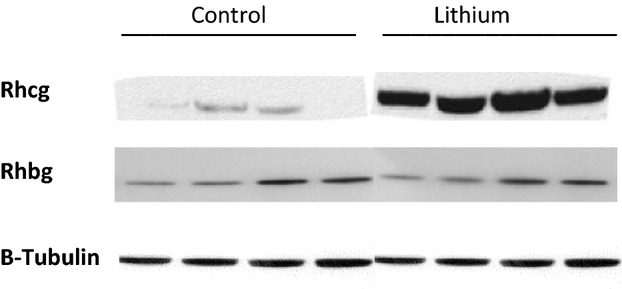
Effect of lithium treatment on expression of the ammonia transporter family members, Rhcg and Rhbg, in rat kidney. Top panel shows immunoblot of Rhcg protein expression in the outer medulla of control and lithium‐treated rats. Center panel shows immunoblot of Rhbg protein expression in the outer medulla of control and lithium‐treated rats. Lower panel shows immunoblot of equal loading of protein onto the gel as detected with β‐tubulin. Chronic lithium treatment results in significant increases in Rhcg protein expression in the outer medulla.

Because lithium treatment increased urinary ammonia excretion so dramatically, approximately ninefold over that observed in control rats, the effect of lithium treatment, on the expression of the ammonia transporter family member, Rhcg, was examined. Western blot analysis showed that Rhcg protein was similar in cortical and inner medullary extracts from the kidneys of the lithium‐treated rats as compared to that in control rats. In the outer medulla, however, Rhcg expression was dramatically increased in the lithium‐treated rats (Fig. 5, Table 4). Thus, the increased ammonia excretion with lithium treatment was associated with increased Rhcg expression in the outer medulla.

**Table 4.** Densitometric semiquantitative assessment of both Rhcg and Rhbg in the kidneys of control rats and rats maintained for 6 months on lithium.

Antibody	Control	6‐month lithium	*P* value
Rhcg papilla	100±42	67±19	NS
Rhcg outer medulla	100±37	154±17	<0.001
Rhcg cortex	100±41	71±10	NS
Rhbg papilla	100±37	158±62	NS
Rhbg outer medulla	ND	ND	
Rhbg cortex	ND	ND	

ND, not detectable; NS, not significant.

### Immunohistochemistry

**Figure 6.**
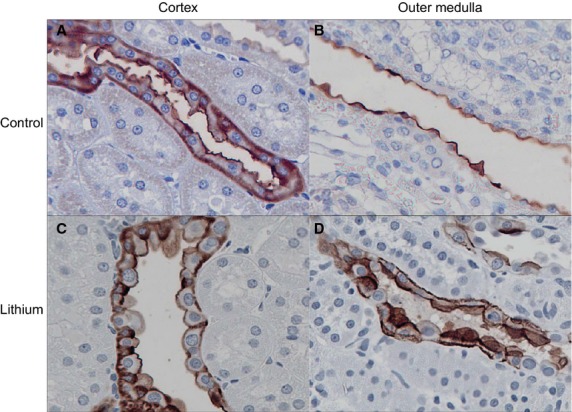
Immunohistochemical localization of Rhcg in renal tissue of control and lithium‐treated rats. (A) In the cortex of control rats, Rhcg is primarily apical in cells in the collecting duct and distal tubules. Basolateral Rhcg immunolabel is also present, but is less intense. (B) In the outer medulla of control rat kidneys, collecting duct cells primarily shown apical Rhcg immunolabel. (C) The cortex of lithium‐treated rats appears similar to that of the controls in its staining pattern. Rare cells without detectable Rhcg immunolabel, likely type B intercalated cells, protrude into the lumen of the collecting duct. (D) In the outer medulla of the lithium‐treated rats, all cells have pronounced basolateral staining, and some cells, thought to be intercalated cells are intensely stained. Magnification 400×.

The results of immunohistochemical staining for the presence of Rhcg are shown in Figure 6. In the control rats, the distribution is consistent with that described in earlier studies. In the cortex staining is principally in the apical membranes of cells in the cortical collecting duct presumed to be intercalated cells. This pattern is continued through the medulla. In contrast, the distribution of Rhcg in the lithium‐treated animals was patchy. In the dilated cortical collecting ducts, some regions show no staining at all, while others show staining of intercalated cells between greatly expanded principal cells. The difference between the two groups is more marked in the outer medulla, where the cells of the collecting duct show intense apical and basolateral staining.

**Figure 7.**
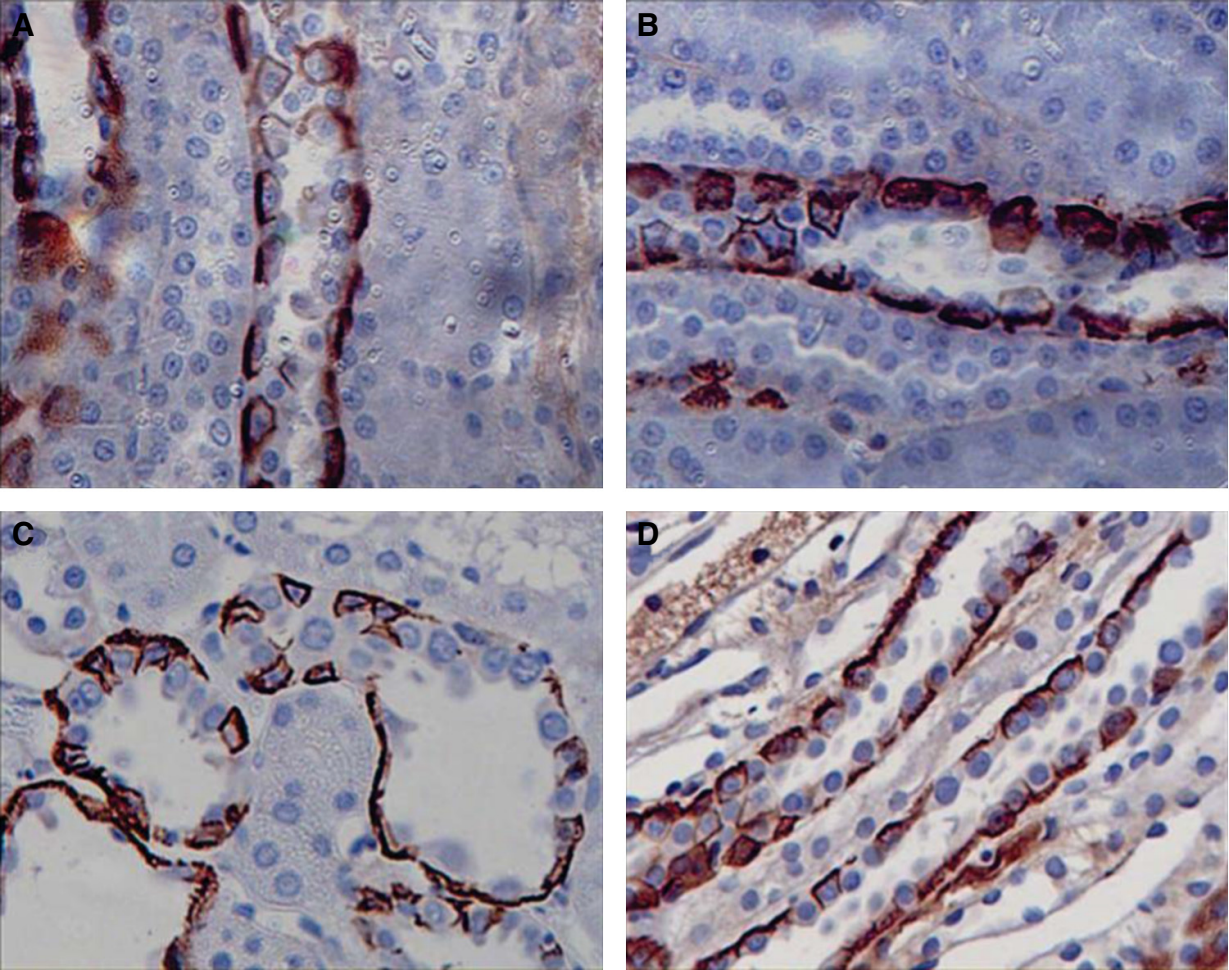
Immunohistochemical localization of RhBg in renal tissue of control and lithium‐treated rats. In the cortex of control rats (A) RhBg is located at the basolateral margins of some cells, presumed to be intercalated cells, in the cortical collecting ducts. A similar pattern and intensity of staining is shown in the cortical collecting ducts of the lithium‐treated animals. In the outer medulla of both control animals (C) and lithium‐treated animals (D) some cells thought to be intercalated cells show a similar pattern of strong basolateral staining. Magnification 400×.

The distribution of the other renal ammonia transporter family member, Rhbg, was also examined. Rhbg was invariably confined to the basolateral membrane of intercalated cells of the distal tubular regions (Fig. 7). There was no significant difference in Rhbg labeling evident by immunohistochemistry.

## Discussion

This study shows that chronic lithium administration has multiple effects on renal ammonia and citrate excretion. In humans, these effects include increased basal and acidosis‐stimulated ammonia excretion. This increase in ammonia excretion was independent of urine pH, suggesting stimulation of ammonia transport mechanisms. Similar findings were observed in rats, which, in addition, also exhibited increased urinary citrate excretion. Finally, in rats, we observed increased expression of the ammonia transporter family member, Rhcg, suggesting that increased Rhcg‐mediated ammonia secretion contributes to the stimulation of renal ammonia excretion by lithium. These findings have important implications for our understanding of acid–base homeostasis in those individuals being treated with lithium.

The first major finding in this study is that chronic lithium exposure in both humans and rats results in increased renal ammonia excretion. In humans, ammonia excretion under baseline conditions was increased approximately threefold in chronic lithium‐treated subjects as compared to that in control subjects. In rats treated with lithium for 6 months, an increase was observed, although quantitatively it was significantly greater. The duration of exposure to lithium necessary to result in increased ammonia excretion remains unknown at present. At least one study, which also reported increased ammonia excretion response to chronic lithium exposure, utilized only a 4‐week exposure (Kim et al. 2003). Thus, these results indicate that chronic lithium exposure is associated with increased ammonia excretion.

The polyuria associated with lithium‐induced nephrogenic diabetes insipidus is unlikely to explain the increased urinary ammonia excretion observed in response to chronic lithium exposure. A previous study examined the effect of polyuria on ammonia excretion in another model of diabetes insipidus, the Brattleboro rat, albeit a model of central diabetes insipidus. Reversing the polyuria, by administering the vasopressin V2‐receptor analogue, 1‐desamino‐8‐D‐arginine vasopressin (dDAVP), increased urinary ammonia excretion (Amlal et al. 2006). This effect of dDAVP was unlikely to be due to effects on intercalated cell‐mediated ammonia transport; the V2 receptor is not expressed in intercalated cells (Carmosino et al. 2007). Lastly, we are aware of no studies reporting the effect of nephrogenic diabetes insipidus or primary polydipsia on ammonia excretion. Thus, polyuria due to a lack of collecting duct water reabsorption appears unlikely to mediate the increased ammonia excretion observed in this study.

The age of the lithium‐treated human subjects was greater than the age of the control subjects, but this is unlikely to explain the differences in ammonia excretion. Several studies have shown that increased age is associated with no impact on basal ammonia excretion and that following an acid load it is associated with a slight impairment of ammonia excretion (Adler et al. 1968; Agarwal and Cabebe 1980; Schuck et al. 1989). Accordingly, the age difference between the lithium‐treated subjects and control subjects in this study is unlikely to explain the differences in ammonia excretion. Moreover, in age‐matched rodents, lithium induced a similar increase in both basal and acidosis‐stimulated ammonia excretion. Accordingly, the results presented in this report strongly support an effect of lithium on renal ammonia metabolism.

Increased ammonia excretion in response to chronic lithium exposure probably involves a variety of mechanisms. In this study, we demonstrated increased expression of the ammonia transporter family member, Rhcg, throughout the collecting duct. Rhcg is known to be necessary for both normal basal ammonia excretion and the increase in ammonia excretion in response to the acute metabolic acidosis. Rhcg is an NH_3_‐specific transporter, which is expressed in both apical and basolateral plasma membranes of collecting duct cells and contributes directly to transcellular collecting duct ammonia secretion (Wagner et al. 2009; Weiner and Verlander 2010, 2011; Nakhoul and Hamm 2013). In multiple conditions of altered ammonia excretion, including metabolic acidosis, reduced renal mass, hypokalemia, cyclosporine‐induced renal tubular acidosis, and ischemia reperfusion injury, there are parallel changes in Rhcg expression (Seshadri et al. 2006a,b; Han et al. 2007, 2011; Kim et al. 2007; Lim et al. 2008). Genetic deletion of Rhcg is known to impair both basal ammonia excretion and the normal response to acid loads (Biver et al. 2008; Lee et al. 2009, 2010). Thus, the lithium‐induced increase in Rhcg expression is likely to mediate an important role in the increased ammonia excretion observed.

Increased ammonia excretion in response to lithium is likely to involve parallel changes in other components of renal ammonia metabolism. Collecting duct ammonia secretion involves parallel transport of NH_3_ and H^+^ (Knepper et al. 1984, 1985; Hamm et al. 1985; Star et al. 1987a,b; Dafnis et al. 1992; Flessner et al. 1992). At least two families of proteins are involved in collecting duct H^+^ secretion, vacuolar H^+^‐ATPase, and P‐type H^+^‐K^+^‐ATPase. Chronic lithium administration is known to increase vacuolar H‐ATPase expression and activity (Kim et al. 2003; Christensen et al. 2011). Whether lithium has effects on the P‐type ATPase, H^+^‐K^+^‐ATPase‐mediated H^+^ secretion remains unknown currently. Nevertheless, this parallel increase in vacuolar H^+^‐ATPase expression, in conjunction with increased Rhcg‐mediated NH_3_ transport, can enable increased transepithelial ammonia secretion in the absence of a change in luminal pH.

Chronic lithium exposure appears to have multiple cellular effects that contribute to its stimulation of renal ammonia excretion. First, lithium exposure increases the volume density of mitochondria in intercalated cells (Ottosen et al. 1987). Because mitochondrial‐generated ATP is necessary for proton secretion, this increase in mitochondrial density is consistent with lithium‐stimulated proton secretion in parallel with NH_3_ secretion. Chronic lithium exposure is known to have prominent effects on the collecting duct cellular composition, causing an increased number of intercalated cells (Christensen et al. 2004), and, as shown recently, evidence of proliferation of principal cells with subsequent conversion into intercalated cells (Christensen et al. 2006; Trepiccione et al. 2013). Because intercalated cells are the primary mechanism of collecting duct ammonia secretion, increased numbers of intercalated cells are consistent with, and likely contribute to, increased collecting duct ammonia secretion. Accordingly, lithium appears to have multiple, interrelated effects which contribute to increased ammonia excretion, including increased expression of Rhcg and H^+^‐ATPase, increased mitochondrial density in collecting duct intercalated cells and increased number of intercalated cells.

It is important to recognize that the studies in the current report reflect effects of chronic lithium exposure. Numerous studies show that acute lithium exposure is associated with impaired urine acidification and collecting duct proton secretion (Homer and Solomon 1962; Roscoe et al. 1976; Nascimento et al. 1977; Arruda et al. 1980; Bank et al. 1982; Dafnis et al. 1992). To some extent, these may be related to the effects of lithium in impairing electrogenic, ENaC‐mediated sodium reabsorption and thereby decreasing development of the luminal negative voltage necessary for normal rates of proton secretion. However, acute lithium exposure also inhibits H^+^‐ATPase enzymatic activity (Dafnis et al. 1992) and it inhibits H^+^‐K^+^‐ATPase activity in the cortical collecting duct, but not in the outer medullary collecting duct (Eiam et al. 1993). Thus, it is important to differentiate explicitly between the acute and chronic effects of lithium on renal acid–base transport.

Another finding in this study is that chronic lithium exposure increases basal rates of citrate excretion in rats, but not in humans. These observations are consistent with previous reports that lithium increases increased citrate excretion in the rat (Bond and Jenner 1974), but not in humans (Bond et al. 1972). Increased citrate excretion with lithium exposure in rats probably has two important implications. First, citrate excretion, as a form of organic anion excretion, contributes to acid–base regulation. Thus, at least in rats, increased citrate excretion counterbalances increased ammonia excretion, and facilitates maintenance of normal acid–base homeostasis. It is important to note, however, that the quantitative role of citrate excretion in net acid excretion is less than 5% of that of ammonia. Second, urinary citrate can complex with urinary calcium, and thereby decreasing the risk of calcium crystallization and development of nephrolithiasis. Lithium therapy is also associated with both primary hyperparathyroidism and with generalized increases in plasma calcium (Garfinkel et al. 1973; Franks et al. 1982; Bendz et al. 1996). The increased urinary citrate excretion may, by complexing with urinary calcium, minimize the risk of calcium‐containing renal stone formation. The mechanism of this increase in rats may involve lithium directly inhibiting the proximal tubule apical citrate transporter, NaDC1. Lithium has been shown to inhibit citrate transport in rabbit brush border membrane vesicles (Wright et al. 1982) and lithium can function as a competitive inhibitor of both rabbit and mouse NaDC1 dicacrboxylate transport, probably by competing for one of the sodium‐binding sites (Pajor 1995; Pajor and Valmonte 1996; Pajor et al. 1998; Pajor and Sun 2000). The differences between human and rats in the effect of lithium on citrate excretion may reflect differences in the affinity of NaDC1 for lithium. In particular, lithium has been reported to not alter human NaDC1 citrate transport significantly (Pajor and Sun 1996; Kahn and Pajor 1999).

Acute metabolic acidosis in humans did not acutely alter citrate excretion. Under chronic conditions, metabolic acidosis is well known to decrease citrate excretion (Simpson 1983; Hamm and Simon 1987; Kaufman and Kahn 1988; Brown et al. 1989; Warden et al. 2010). At least in part, these changes are due to altered apical membrane citrate transport involving altered expression of NaDC‐1 (Jenkins et al. 1985; Aruga et al. 2000). The general time course of changes in protein expression, requiring multiple hours, and even days, would minimize adaptive changes in protein expression during the time course of the acute studies reported in this manuscript. A second mechanism by which chronic metabolic acidosis is thought to alter citrate excretion is through a higher H^+^ concentration in the luminal fluid in the proximal tubule, resulting in protonation of citrate^−3^ to form citrate^−2^. Because citrate^−2^ is the transported form of citrate, this is proposed to increase citrate reabsorption, and thus contribute to decreased citrate excretion (Hamm and Simon 1987; Weiner and Verlander 2012, 2013). However, the lack of change in citrate excretion with acute metabolic acidosis, despite significant changes in systemic pH, systemic bicarbonate in urine pH, in this study suggests that this latter mechanism is likely to be a quantitatively less important regulatory mechanism, at least in humans under the conditions studied in this manuscript.

Lithium‐treated subjects in this study had identical serum bicarbonate concentrations despite the increase in ammonia excretion. This suggests a parallel increase in alkali utilization, either excretion or incorporation into body tissues. Chronic lithium administration is associated with increased bone density and resultant decrease in bone fracture rates (Adler et al. 1968; Agarwal and Cabebe 1980; Schuck et al. 1989; Vestergaard et al. 2005; Wilting et al. 2007; Bolton et al. 2008; Zamani et al. 2009; Warden et al. 2010). Bone formation and mineralization requires parallel alkali incorporation into the bone matrix. Thus, this increased bone formation likely, at least in part, contributes to the normal acid–base balance observed. There is also likely to be increased urinary bicarbonate excretion. Although urine pH, and thus likely urinary bicarbonate concentration, did not differ between lithium‐treated subjects and control subjects, the higher urine volume associated with the mild nephrogenic diabetes insipidus indicates that urinary bicarbonate excretion was also greater in lithium‐treated subjects. However, renal bicarbonate losses are unlikely to be the primary etiology of increased ammonia excretion as serum bicarbonate were not lower as would have been expected if renal bicarbonate excretion had induced a mild metabolic acidosis that led to the increased ammonia excretion. Finally, we cannot exclude changes in organic anion excretion in stool that might be induced by lithium and thereby also contribute to compensation of systemic acid–base homeostasis.

In summary, the current studies provide important new insights into the effects of chronic lithium exposure on renal acid–base homeostasis. In both humans and rats, ammonia excretion is increased, both basal ammonia excretion and the response to an exogenous acid load. This increased ammonia excretion is likely to involve increased Rhcg‐mediated NH_3_ transport, described for the first time in this report, in addition to previous findings of increased H‐ATPase expression and increased number of acid‐secreting, A‐type intercalated cells. In addition, chronic lithium administration in rats increases citrate excretion. Thus, chronic lithium exposure has substantial effects on renal acid–base excretion.

## Acknowledgments

We thank Dr Sam Lucas, Department of Physiology, University of Otago (now University of Birmingham, UK) for assistance with the blood gas analysis.

## Conflict of Interest

No conflicts of interest, financial or otherwise, are declared by the author(s).
